# P02.105. Short term improvement of subject well-being after a single Rhythmical Massage: a prospective, randomized, controlled trial

**DOI:** 10.1186/1472-6882-12-S1-P161

**Published:** 2012-06-12

**Authors:** J Kanitz, C Rihs, I Krause, M Reif, G Henze, G Seifert

**Affiliations:** 1 Charité, University of Medicine, Berlin, Berlin, Germany; 2 Institut for Clinical Research (IKF Berlin), Berlin, Germany

## Purpose

Rhythmical Massage (RhM) is an enlargement of the classical manual massage and has been created by Dr. Ita Wegman at the beginning of the 20th century according to the principles of Anthroposophic Medicine. In addition to effects on the skin and muscles, RhM is believed to have both general effects (e.g. enhancing physical vitality) and disease-specific effects (e.g. internal medicine, orthopedics, neurology, pediatric, rehabilitation). The goal of this randomised, single-blinded study was to assess the efficacy of a single RhM intervention either with aromatic oil (RA), neutral oil (RM) or a sham massage (SM) on subject well-being.

## Methods

101 healthy adults (mean age: 25.2; SD: 4.7) were randomised to one of three groups (RM, RA or SM). All participated in the Trier Social Stress Test (TSST) before receiving a single massage intervention of about 60 minutes including 20 minutes of quiet time. Well-being was assessed by standardised questionnaires (MDBF, BF-S, B-L) and visual analogue scales (VAS) prior to the beginning of the study and afterwards. Additionally, salivary cortisol was measured.

## Results

After a single Intervention, the RM and the RA group showed statistically significant improvements compared to the SM group in the dimensions of mood and alertness (MDBF), VAS‚ emotional state, and relaxation of neck and shoulders. No difference was found between the RM and the RA group. Salivary Cortisol, BF-S and B-L scales did not differ significantly between the three groups over time. All participants had comparable expectations concerning their participation and no one had previously experienced RhM.

## Conclusion

One single RhM intervention leads to a better mood, alertness and relaxation that is not explainable from the setting, because no improvement was found in the SM group. No additional effects were found for the aroma oil.

